# The regulatory pathways of distinct flowering characteristics in Chinese jujube

**DOI:** 10.1038/s41438-020-00344-7

**Published:** 2020-08-01

**Authors:** Xianwei Meng, Ying Li, Ye Yuan, Yao Zhang, Hongtai Li, Jin Zhao, Mengjun Liu

**Affiliations:** 1 Research Center of Chinese Jujube, Hebei Agricultural University, Baoding, 071000 China; 2 College of Life Science, Hebei Agricultural University, Baoding, 071000 China

**Keywords:** Transcription, Plant development

## Abstract

Flowering is the most important event in higher plants. Compared to most fruit tree species, Chinese jujube (*Ziziphus jujuba* Mill.), the most important member of the large, diverse Rhamnaceae family and a leading dry fruit-producing species, has unique characteristics that include a short juvenile phase and extremely fast flower bud differentiation. However, the distinct mechanism of flowering regulation in Chinese jujube is still unclear. The morphological and cytological development period of jujube flowering was first investigated, and the crucial developmental stages were defined. Flower bud differentiation in Chinese jujube took only approximately 11–13 days, which is a distinct characteristic of perennial fruit trees. Afterward, 44 genes related to six flowering pathways were identified in the jujube genome and were found to be randomly distributed among 11 of the 12 chromosomes. Tissue-specific and spatiotemporal expression patterns showed that all these genes were expressed in the flowers. Overall, photoperiod-related genes were highly expressed during flower bud differentiation. These genes were also positively responsive to photoperiod regulation and phase change processes, indicating that photoperiod- related genes play crucial roles in jujube flower bud differentiation. Under protected cultivation, *ZjPIF4*, a temperature-related gene, was expressed in the early stages of flowering and responded to increasing temperatures. Moreover, STRING analysis and yeast two-hybrid screening indicated that photoperiod-related (ZjCO) and temperature-related (ZjPIF4) proteins could interact with ZjFT, the key protein involved in the determination of flowering time, indicating crosstalk between photoperiod-related pathways and ambient temperature-related pathways in jujube. This study is the first report to comprehensively analyze the flowering pathways in Chinese jujube and revealed that photoperiod-related and ambient temperature-related pathways are the main mechanisms regulating the distinct flowering process and that members of the ZjPHY family (ZjPIF4, ZjFT, and ZjCO5) are the key factors involved in the regulatory network. These results will increase our understanding of the molecular and genetic mechanisms of flowering in Chinese jujube and provide meaningful clues for the flowering regulation of other fruit tree species.

## Introduction

The flowering process in higher plants is the end result of a central hub controlling the transformation from the vegetative growth stage to the reproductive growth stage. Flowering time has an important impact on the breeding, early fruiting and high yields of perennial woody fruit trees. An intricate network involving various (epi-) genetic regulators controls the timely onset of flowering. A series of genes related to flowering have been isolated and identified from model plant species and woody fruit tree species^1,2^.

Flowering involves complex regulatory mechanisms and pathways that ensure flower transformation at the appropriate time. In Arabidopsis, there are mainly six regulatory pathways, i.e., the vernalization-related, photoperiod-related, gibberellin-related, autonomous-related, ambient temperature-related, and age-related pathways^3^. These pathways are independent of each other but exhibit crosstalk to form a flower regulatory network with precise regulatory functions^4^. According to previous studies, the flowering process is affected by external environmental conditions and internal growth and development. However, the flowering pathways of most fruit tree species are still unclear.

Chinese jujube (*Ziziphus jujuba* Mill.) is the most important member of the large, diverse Rhamnaceae family and one of the most economically important fruit tree species in China^5^. In general, most fruit trees have long juvenile vegetative phases. Compared to most other fruit trees, Chinese jujube trees have a very short juvenile phase and the ability to produce fruit within the first year of planting, with flower bud differentiation and fruiting occurring in the same year. In our previous study, several MADS-box genes related to flowering were identified^6^. However, there have been no reports on the jujube flowering pathway. Therefore, to clarify the main pathways regulating jujube flowering and the critical genes involved in jujube flowering, the morphology and developmental period of jujube flowers were observed. In addition, the genes related to different flowering pathways were identified at the genomic level, and their involvement in the flowering process was further analyzed. These results will increase our understanding of the molecular and genetic mechanisms underlying flowering in Chinese jujube and provide clues for flowering regulation in other fruit tree species.

## Results

### Morphological and cytological observations of flower development in Chinese jujube

**Fig. 1:**
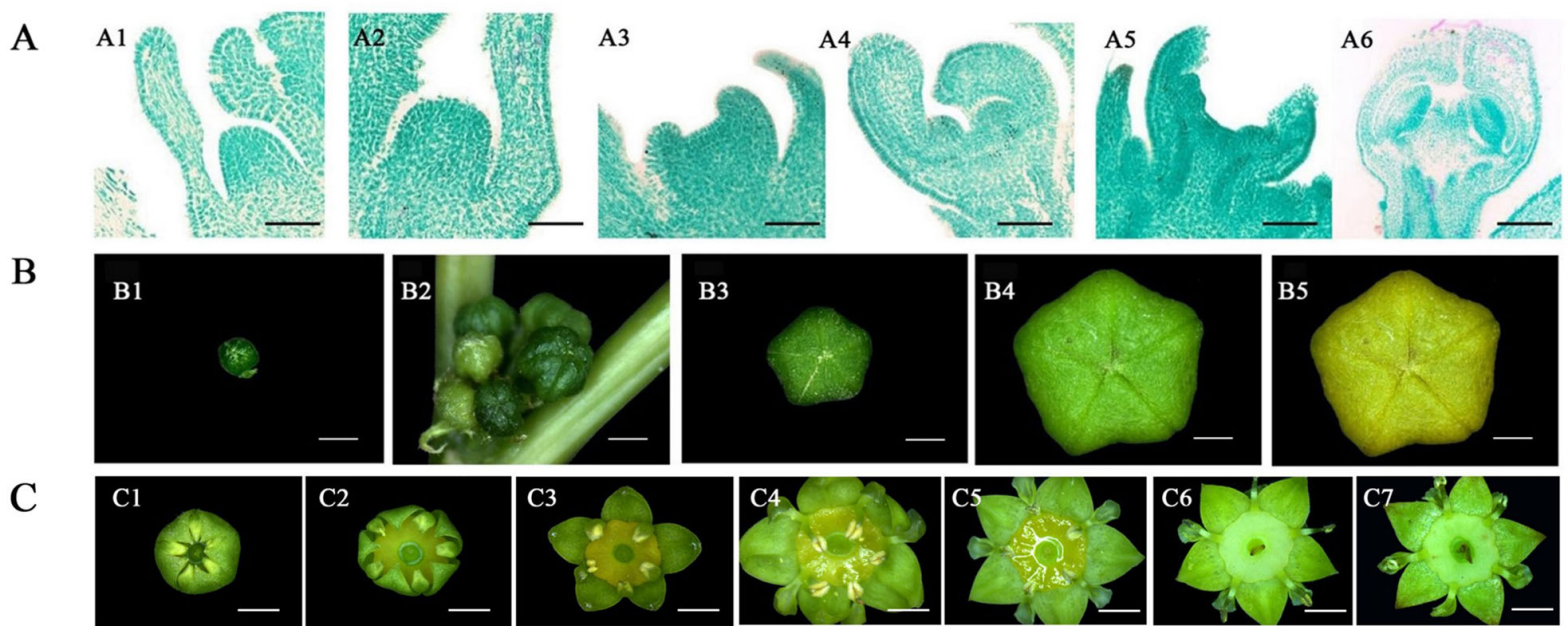
The flowering process of Chinese jujube (*Ziziphus jujuba* Mill. “Dongzao”). **a** (Flower bud differentiation). A1: Undifferentiated period; A2: Initial stage; A3: Sepal differentiation; A4: Petal differentiation; A5: Stamen differentiation; A6: Carpel differentiation; Bar: 50 µm. **b** (Flower development). B1: Bud emergence; B2: Inflorescence emergence; B3: Bud flattening; B4: Bud swelling; B5: Bud yellowing. Bar: 500 µm. **c** (Flower opening). C1: Bud break; C2: Early blooming; C3: Semiopening; C4: Petal elongation; C5: Petal flattening; C6: Stamen flattening; C7: Wilting; Bar: 1000 µm

To determine the critical sampling periods, we systematically observed the entire flowering process of Chinese jujube. In general, there are three stages during the plant flowering process: sex determination, flower bud differentiation, and floral organ development. From sex determination to flower bud differentiation, we divided the process into six stages at the histological level: the undifferentiated stage, initial flower bud differentiation, sepal differentiation, petal differentiation, stamen differentiation, and carpel differentiation (Fig. 1a). Correspondingly, the morphology and developmental period of the bearing shoots were also investigated (Table S1).

At the undifferentiated stage, two bracts appeared on both sides of the leaf axils, and no obvious convex apical meristem was observed (Fig. 1A1, Table S1). When flower bud differentiation began, the top of the bud gradually flattened to a pointed circle, forming a flower primordium (Fig. 1A2). It took approximately 1.5–2 days for the transition from the initial stage to sepal differentiation. Compared to that of other stages, differentiation from sepals to petals was the longest stage (approximately 6–7 days, Table S1). The protuberance became a rudimentary dichotomous stigma (Fig. 1A6), and a flower bud was visible between the leaf axils. In total, the whole process of flower bud differentiation in Chinese jujube took only approximately 11–13 days, and the extremely fast differentiation of flowers is a distinct characteristic of perennial fruit tree species.

Flower development began with the enlargement of the flower bud. Accordingly, we divided flower development into five stages: bud emergence, inflorescence emergence, bud flattening, bud swelling, and bud yellowing (Fig. 1b). With the development of flower buds in bearing shoots, the bud became lighter in color. When the diameter of the bud reached approximately 2600 µm, the bud shape changed from round to flat. The bud color ultimately became yellow-green or light-yellow (Fig. 1b). The bud development period of Chinese jujube lasted 25–34 days.

We also observed the single-flower opening process of Chinese jujube and divided it into seven stages: bud splitting, early opening, sepal flattening, the petal upright stage, petal flattening, stamen flattening, and wilting (Fig. 1c). The single-flower opening process was short and took only approximately 2–3 days.

### Identification of candidate genes related to jujube flower bud differentiation

**Fig. 2:**
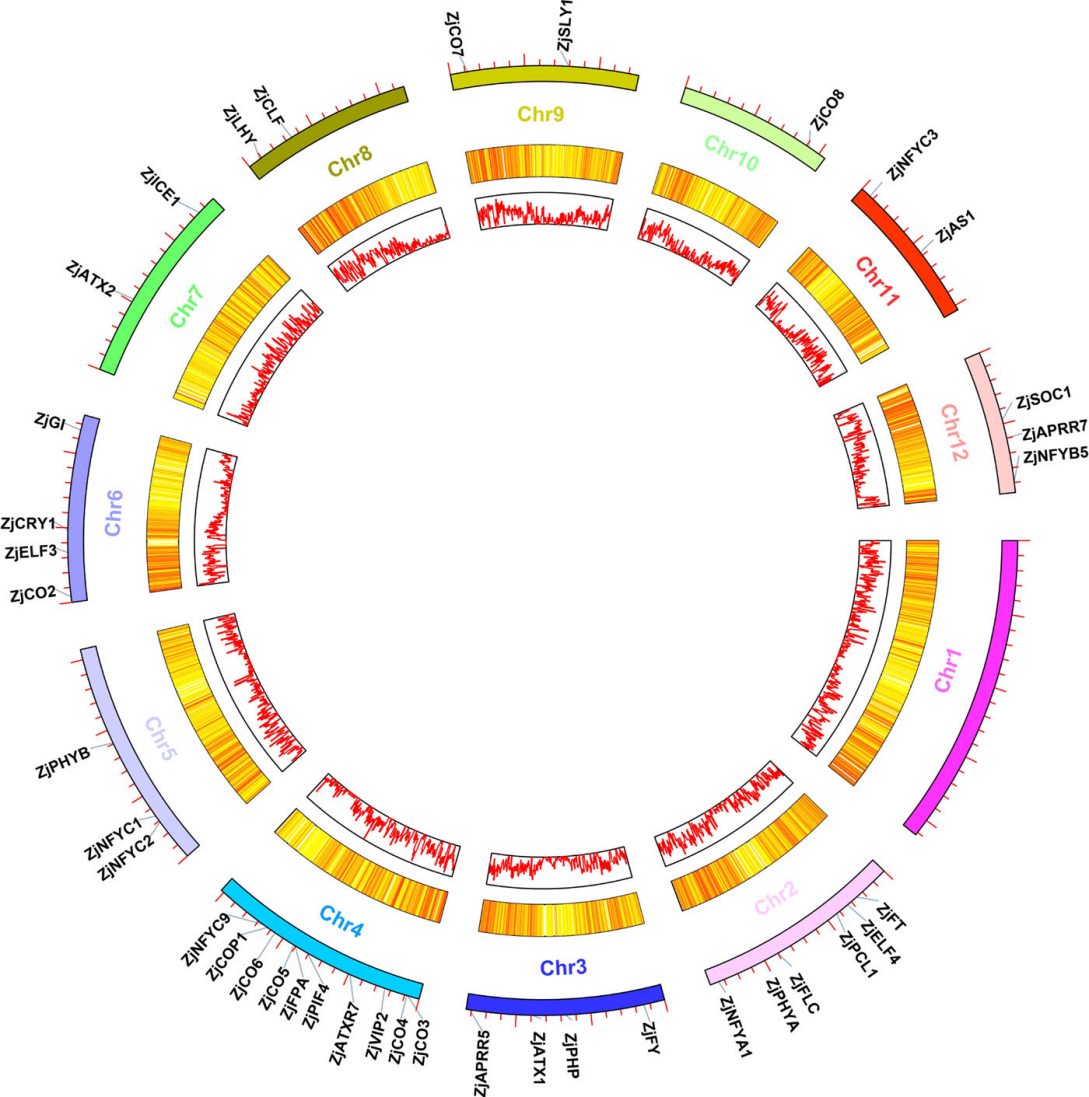
Positions of the flowering-related genes on the jujube chromosomes. (In the circle diagram, the gene density is represented by two circles from inside to outside, and the outer circle shows the gene position on the chromosome.)

After performing a homology search, we identified 44 candidate genes related to flower bud differentiation for six flowering pathways in Chinese jujube (Table 1). The ORF of these genes ranged from 222 to 3900 bp, and their encoded proteins ranged from 83 to 1299 amino acids (aa), with a predicted molecular mass of 8.16–144.81 kDa and a pI ranging from 4.34 to 10.81. Moreover, these genes were mapped to the 11 pseudochromosomes (Fig. 2). Most members of the CONSTANS family were concentrated on Chromosome (Chr) 4, but *ZjCO2* and *ZjCO8* were distributed on Chr6 and 10, respectively. We also compared the homologous genes between jujube and other related species, and the results showed that most identified flowering-related genes had high homology with those of other species (Table S2), indicating that conserved evolutionary pathways are shared among these plant species.

**Table 1:** Information on genes related to jujube flower bud differentiation in Chinese jujube

Gene name	Gene ID	Chr	ORF (bp)	Size (aa)	MW (kDa)	pI	Exon number	Pathway
*ZjCO1*	XM_016012995.1	Un	1167	388	42.48098	5.51	2	Photoperiod
*ZjCO2*	XM_016028783.1	Chr6	1020	339	37.38886	5.81	2	
*ZjCO3*	XM_025072777.1	Chr4	1110	369	39.71734	5.94	3	
*ZjCO4*	XM_016023535.1	Chr4	252	83	9.70229	10.81	2	
*ZjCO5*	XM_016041736.1	Chr1	786	261	28.57188	4.34	2	
*ZjCO6*	XM_016025616.1	Chr4	1284	427	46.70826	5.37	2	
*ZjCO7*	XM_016036150.1	Chr9	1200	399	45.06296	5.32	5	
*ZjCO8*	XM_016040126.1	Chr10	1410	469	51.9196	5.59	3	
*ZjCOP1*	XM_016025733.1	Chr4	2022	673	75.93132	7.29	13	
*ZjGI*	XM_016030871.1	Chr6	3546	1181	129.56633	6.18	16	
*ZjPHYA*	XM_016019337.1	Chr2	3393	1130	125.10612	6.12	5	
*ZjPHYB*	XM_016027703.1	Chr5	3393	1130	125.72442	5.85	5	
*ZjPHYC*	XM_016045282.1	Un	3369	1122	125.03431	5.67	6	
*ZjCRY1*	XM_016029911.1	Chr6	2037	678	76.63191	5.52	5	
*ZjAS1*	XM_016041509.1	Chr11	1071	356	40.98776	9.49	4	
*ZjICE1*	XM_016032924.1	Chr7	1650	549	59.80445	4.96	4	
*ZjNFYA1*	XM_016020414.1	Chr2	1047	348	37.89429	6.18	6	
*ZjNFYB3*	XM_016016939.1	Un	390	129	13.82761	5.70	3	
*ZjNFYB5*	XM_016044495.1	Chr12	414	137	15.34737	5.67	1	
*ZjNFYC1*	XM_016026661.1	Chr5	711	236	25.83705	5.17	2	
*ZjNFYC2*	XM_016026566.1	Chr5	822	273	30.75376	5.88	4	
*ZjNFYC3*	XM_016019708.1	Chr2	786	261	29.01577	5.89	3	
*ZjNFYC9*	XM_016032789.1	Chr7	777	258	28.83348	5.96	4	
*ZjPHP*	XM_016021799.1	Chr3	1347	448	48.83805	9.16	5	Vernalization
*ZjVIP2*	XM_016027633.1	Un	816	271	29.8654	5.68	4	
*ZjATX1*	XM_016022022.1	Chr3	222	73	8.15652	6.71	3	
*ZjATXR7*	XM_016024470.1	Chr4	3900	1299	144.81003	8.60	17	
*ZjATX2*	XM_016031671.1	Chr7	3396	1131	127.46562	6.93	26	
*ZjEMF2*	XM_016046518.1	Un	780	259	29.93849	6.36	11	
*ZjCLF*	XM_016034323.1	Chr8	2778	925	103.52513	8.57	17	
*ZjMSI1*	XM_016045216.1	Un	1272	423	48.2669	4.74	8	
*ZjFLC*	XM_016019142.1	Chr2	828	275	30.68936	6.65	5	
*ZjPIF4*	XM_016025115.1	Chr4	1464	487	53.62186	6.38	7	Ambient temperature
*ZjAPRR7*	XM_016044067.1	Chr12	2361	786	85.8622	8.65	10	Circadian clock
*ZjAPRR5*	XM_016023239.1	Chr3	2100	699	77.43027	6.72	8	
*ZjLHY*	XM_016033463.1	Chr8	2328	775	85.2531	5.83	6	
*ZjPCL1*	XM_016018524.1	Chr2	987	328	35.54645	5.89	4	
*ZjELF3*	XM_016029616.1	Chr6	2169	722	78.08207	6.34	5	
*ZjELF4*	XM_016021901.1	Chr2	315	104	11.9029	4.91	2	
*ZjFPA*	XM_016025379.1	Chr4	2790	929	103.24512	6.74	4	Autonomous
*ZjFY*	XM_016020808.1	Chr3	2190	729	80.75523	8.72	18	
*ZjSLY1*	XM_016037559.1	Chr9	1824	607	68.20743	5.59	2	GA
*ZjFT*	XM_016018112.1	Chr2	525	174	19.63019	7.75	4	Flowering integration factor
*ZjSOC1*	XM_016043834.1	Chr3	777 |	258	29.60145	9.74	7

### Organ-specific expression of the genes related to jujube flower bud differentiation

**Fig. 3:**
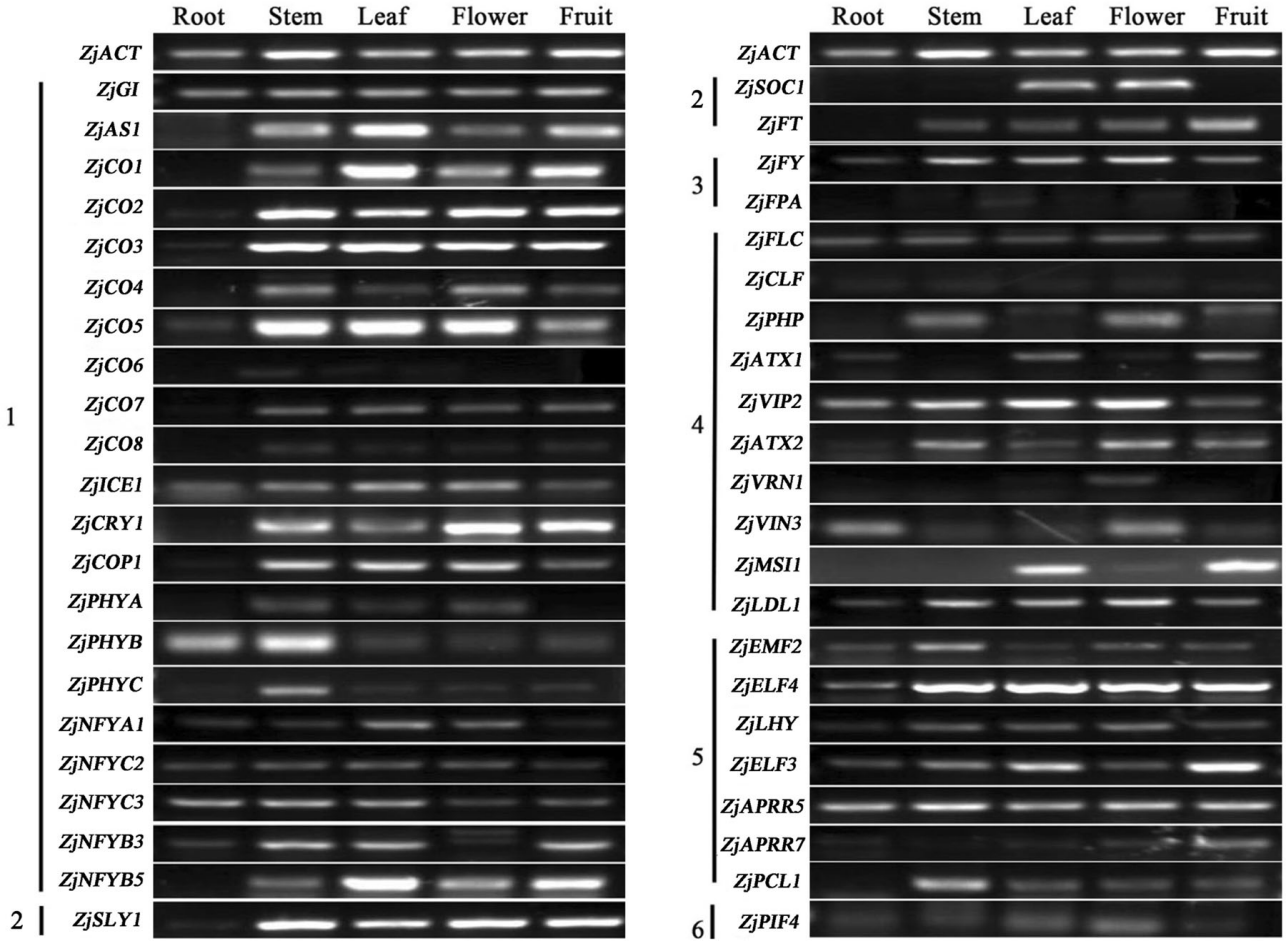
Expression patterns of jujube flowering-related genes in vegetative and reproductive organs by RT-PCR. *ZjACT* was used as an internal control gene. Note: 1-photoperiod, 2-GA and flowering integration factor, 3-autonomy, 4-vernalization, 5-circadian clock and 6-ambient temperature

We investigated the expression patterns of 44 candidate genes in five different organs (Fig. 3), and the results showed that most of them were expressed in both the vegetative and reproductive organs. Compared to that in other organs, the expression of these genes in the roots was lower. In particular, the genes related to the photoperiod pathway were highly expressed in the stems, leaves, and flowers (Fig. 3). The genes showing high expression levels in the stems may be related to flower bud differentiation.

### Photoperiod-related genes may play key roles in jujube flower bud differentiation

**Fig. 4:**
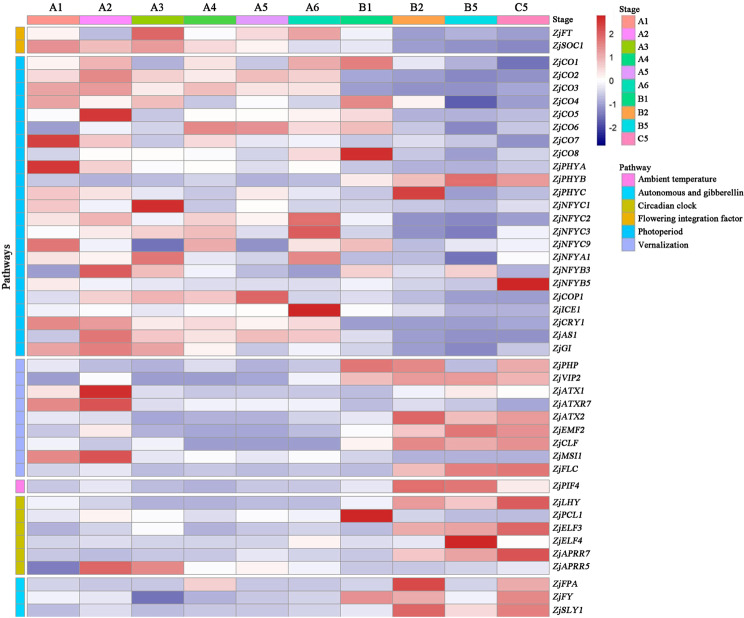
Expression patterns of flower-related genes with different pathways during jujube flower development. *ZjACT* was used as an internal control gene. The mean expression value was calculated from three independent biological replicates. Color bar: Log2(fold change). Note: A1. Unp (Undifferentiated period); A2. Ins (Initial stage); A3. Sed (Sepal differentiation); A4. Ped (Petal differentiation); A5. Std (Stamen differentiation); A6. Cad (Carpel differentiation); B1. Bue (Bud emergence); B2. Ine (Inflorescence emergence); B5. Ybs (Bud yellowing); C5. Pes (Petal flattening). The scaled log2 expression values are shown from blue to red

To further investigate the role of the above-mentioned genes in flower development, their expression patterns were determined throughout the flowering process using qRT-PCR. Based on the jujube flowering process (Fig. 1), ten typical periods were selected in this study. High expression levels of the genes related to the photoperiod pathway and two crucial genes (*ZjFT* and *ZjSOC1*) were observed during the early flower bud differentiation stages (Fig. 4), indicating that these genes may be key genes involved in jujube flowering. The trends of the expression patterns of most genes involved in vernalization and circadian rhythm pathways were opposite those of the genes involved in the photoperiod pathway, meaning that they should regulate the late flowering stages (Fig. 4). Thus, the above results preliminarily suggested that the genes related to the photoperiod pathway might play critical roles in jujube flower bud differentiation.

### Photoperiod-related genes and *ZjPIF4* positively respond to jujube flowering under protected cultivation

To verify the roles of the photoperiod pathway in jujube flowering, jujube trees in the greenhouse were used, and the flowering time was advanced to January from May in an open field. There are four types of shoots in jujube trees: primary shoots, secondary shoots, mother-bearing shoots, and bearing shoots. Mother-bearing shoots, which are extremely condensed (expanding by only approximately 1 mm a year), produce bearing shoots every year. Bearing shoots are the only flowering and fruiting shoots in Chinese jujube and drop in the winter. Thus, the expression of the above genes in mother-bearing shoots and their branch parts (bark) were further investigated before flowering.

**Fig. 5:**
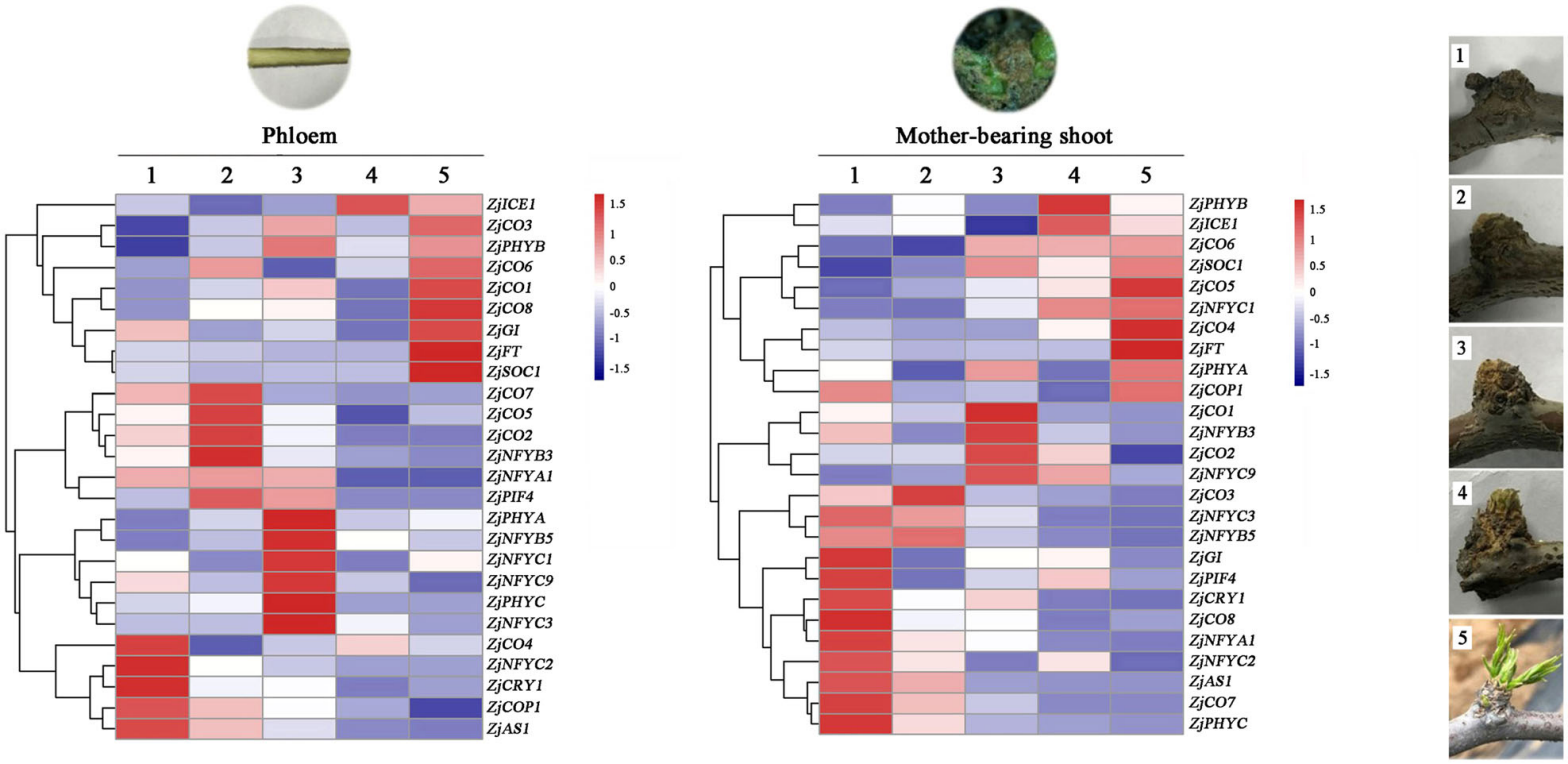
Relative expression of photoperiod genes in jujube mother-bearing shoots under protected cultivation. The mean expression value was calculated from three independent biological replicates. Note: 1. The first stage (October 28, 2018); 2. The second stage (January 8, 2019); 3. The third stage (January 18, 2019); 4. The fourth stage (January 28, 2019); 5. The fifth stage (February 28, 2019). The scaled log2 expression values are shown from blue to red

Most photoperiod-related genes responded differently to the different developmental stages in the mother-bearing shoots and in their base branch bark (Fig. 5). Some of the genes (*ZjICE1*, *ZjCO6*, *ZjSOC1*, and *ZjFT*) in both tissues were expressed at high levels in the later developmental stages, suggesting that these genes may be involved in the flowering process. This experiment also indicated that the photoperiod pathway plays crucial roles in jujube flowering. Compared with that in the base branch bark, the expression of *ZjPIF4* in the mother-bearing shoots was much higher in the early stages, indicating that *ZjPIF4* is also an important regulator of floral initiation.

### Photoperiod-related genes involved in the jujube phase change process

The seedlings of hybrid progeny of JMS2×Xing 16 with different flowering times were observed in two consecutive years. The flowering rates in the first and second years were 20% and 100%, respectively (Table S3). The individual seedlings were divided into juvenile, transition, and adult areas according to the secondary shoot and flowering positions (Fig. S1). The first flowering position of the hybrid progeny was concentrated in transitional secondary shoots, which is expected to become a marker trait of stage transformation.

**Fig. 6:**
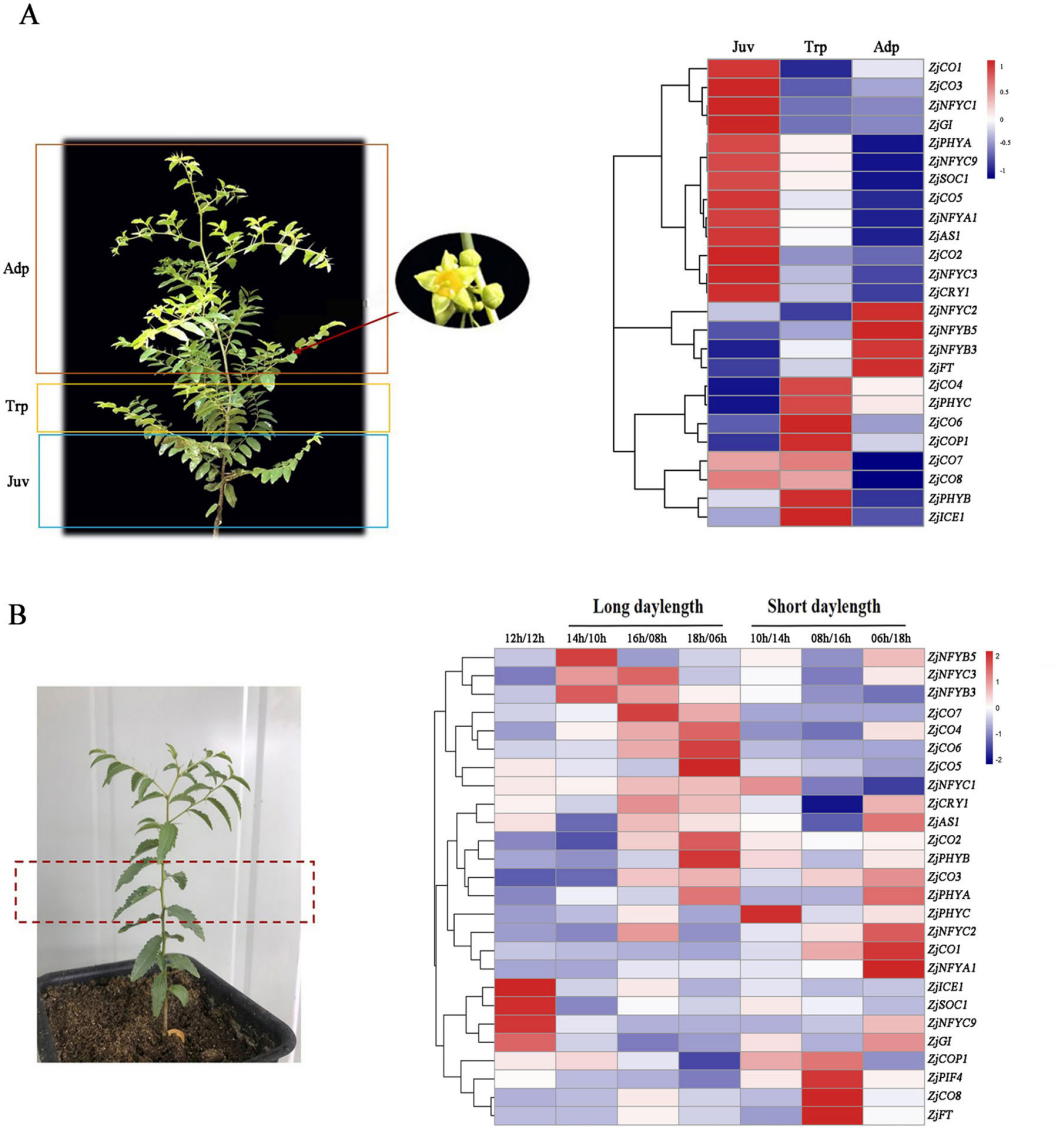
Relative expression of the photoperiod genes of jujube seedlings during the phase change process. **a** Note: 1. Juv (juvenile period); 2. Trp (transition period); 3. Adp (adult period). The scaled log2 expression values are shown from blue to red. **b** Relative expression of the photoperiod genes under long-day and short-day treatments. Note: 1. Control (12 h light/12 h dark); 2. The long-day treatment: 14 h light/10 h dark; 3. The long-day treatment: 16 h light/8 h dark; 3. The long-day treatment: 18 h light/6 h dark; 5. The short-day treatment: 10 h light/14 h dark; 6. The short-day treatment: 8 h light/16 h dark; 7. The short-day treatment: 6 h light/18 h dark. The scaled log2 expression values are shown from blue to red. The mean expression values were calculated from three independent biological replicates

We investigated the growth dynamics of the seedlings and the expression patterns of photoperiod-related genes during the phase transition (Fig. 6a). Most genes were highly expressed in the juvenile period, five genes (*ZjICE1*, *ZjCO6*, *ZjCO4*, *ZjPHYC*, and *ZjCOP1*) were highly expressed in the transition period, and four genes (*ZjNFYC2*, *ZjNFYB5*, *ZjNFYB3*, and *ZjFT*) were highly expressed in the adult period. Overall, the expression patterns of most photoperiod-related genes during the phase transition of seedlings were consistent with those in the perennial tree species (Fig. 4), suggesting that photoperiod-related genes are also involved in the phase transition.

### Photoperiod-related genes positively respond to photoperiod regulation

To further verify the photoperiod-related genes involved in jujube flowering, the jujube seedlings were subjected to long-day and short-day photoperiod treatments under the same growth conditions. We found that 68% and 58% of the genes responded to the long-day and short-day treatments, respectively (Fig. 6b). In the long-day treatment, the expression of these genes (*ZjNFYB5*, *ZjNFYC3*, *ZjNFYB3*, *ZjCO7*, *ZjCO4*, *ZjCO5*, *ZjCO6*, *ZjNFYC1*, *ZjCRY1*, *ZjAS1*, *ZjPHYA*, *ZjCO2*, *ZjCO3*, *ZjNFYC2*, and *ZjCOP1*) increased significantly with increasing light duration, and the highest expression was found under an 18 h/6 h light/dark photoperiod. In the short-day treatment, the expression of these genes (*ZjNFYB5*, *ZjCRY1*, *ZjAS1*, *ZjPHYA*, *ZjCO3*, *ZjNFYC2*, *ZjCO1*, *ZjNFYA1*, *ZjNFYC9*, and *ZjGI*) increased significantly and peaked under a 6 h/18 h light/dark photoperiod. The photoperiod regulation significantly altered the expression of photoperiod-related genes, and the long-day treatment also increased the early flowering of jujube seedlings (Table S4).

### Protein–protein interactions among several key flower-related proteins

**Fig. 7:**
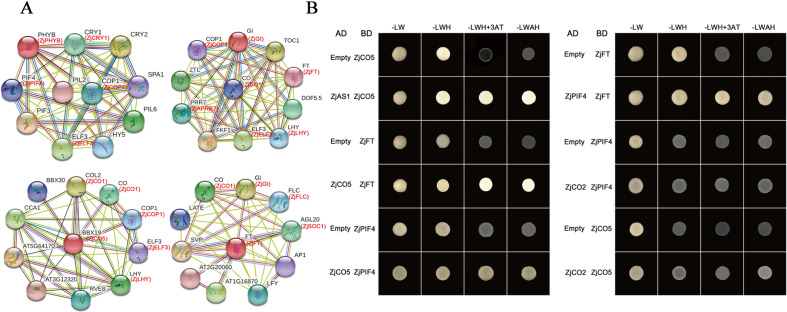
Protein–protein interactions among select photoperiod-related proteins. **a** Protein–protein interaction analysis of ZjPHYB, ZjGI, ZjCO and ZjFT by STRING. b Yeast two-hybrid screening of select proteins involved in the photoperiod-related and ambient temperature-related pathways

Based on the orthologs of Arabidopsis, the interactions of photoperiod-related proteins of Chinese jujube were predicted by STRING analysis (Fig. 7a). The results predicted that many candidate key proteins interact with each other, i.e., PHYB (homolog of ZjPHYB) could interact with PIF4 (homolog of ZjPIF4), CRY1 (homolog of ZjCRY1) could interact with COP1 (homolog of ZjCOP1), COP1 (homolog of ZjCOP1) could also interact with GI (homolog of ZjGI) and CO (homolog of ZjCO1), BBX19 (homolog of ZjCO5) could interact with CO (homolog of ZjCO1) and COP1 (homolog of ZjCOP1), and FT (homolog of ZjFT) could interact with CO (homolog of ZjCO1) and AGL20 (homolog of ZjSOC1). A yeast two-hybrid assay (Y2H) then verified that there were interactions between ZjCO5 and ZjFT and between ZjCO5 and ZjAS1 (Fig. 7b). Furthermore, ZjPIF4, an important regulator related to photoperiod and temperature, interacted directly with ZjCO5 and ZjFT (Fig. 7b). Take together, these results indicated that the photoperiod- and ambient temperature-related pathways are the key pathways involved in jujube flowering and that the ZjPHY family members ZjPIF4, ZjFT and ZjCO5 are the key factors involved in the regulatory network.

## Discussion

### The distinct flowering process of Chinese jujube

Flowering is an intricate developmental process involving several stages, including floral induction, floral initiation, flower development, and blooming^7^. Here, we found that flower bud initiation and differentiation lasted less than 1 month in Dongzao, the most popular jujube cultivar for fresh markets^5^, and showed a fast-slow-fast trend. However, in other fruit tree species, such as apple, pear, and peach, flower bud initiation starts with the cessation of shoot growth^7,8^, and floral differentiation occurs in the next year^9,10^. Thus, flower bud initiation and differentiation occur in two consecutive years in most fruit trees^11^. As a perennial woody fruit tree species, Chinese jujube has a very fast differentiation rate during flowering, which is a distinct trait of fruit trees, but its molecular mechanisms need to be further studied. Additionally, apart from the 44 flowering-related genes identified, additional genes may be involved in the complex flowering process in the jujube genome.

### The photoperiod pathway is the dominant pathway in Chinese jujube flowering

Many plants sense seasonal cues, day length or photoperiod changes to align the timing of the developmental transition to flowering with the changing seasons for reproductive success^12^. In this study, the expression levels of most photoperiod-related genes in jujube were responsive to flower bud differentiation, which was confirmed using samples with phase transitions and those under protected cultivation. The transition from the juvenile vegetative phase to the adult vegetative phase is referred to as the juvenile-to-adult transition or vegetative phase change^13^. This transition is an important shift for the acquisition of adult vegetative characteristics and subsequent reproductive competence^14^. Most photoperiod-related genes were highly expressed in the juvenile and transition phases, indicating that their function contributed to the juvenile-to-adult transition.

In general, the circadian rhythm pathway is associated with the photoperiod pathway, and endogenous circadian rhythms are sensitive to light/dark stimuli^15,16^. The expression patterns of most genes involved in these two pathways showed sequential expression in this study (Fig. 4). Overall, the expression of the genes related to the photoperiod pathway occurred earlier than did that of the genes related to the circadian rhythm pathway, indicating that the photoperiod pathway might play a pivotal role during flower bud differentiation and that the circadian rhythm pathway may play an important role in flower development. Compared to annual herbs, the growth process of perennial fruit trees is more complex, and the environmental factors are more uncontrollable; thus, their molecular mechanisms of flowering might have distinct characteristics and need to be further studied.

*ZjPIF4* is an orthologous gene of PIF4, which is a core component of the photoperiod- and ambient temperature-related pathways^17–19^. Under protected cultivation, the expression of *ZjPIF4* was also high in early developmental stages, and Y2H assays proved that ZjPIF4 could also interact with the photoperiod-related proteins ZjCO5 and ZjFT (Fig. 7b), indicating that crosstalk occurred between the photoperiod- and ambient temperature-related pathways in jujube. Moreover, the expression of an increasing number of photoperiod-related genes increased significantly under long-day conditions, and the jujube seedlings showed early flowering (Fig. 6b, Table S4). Overall, all the above results indicated that the photoperiod pathway is the dominant pathway in Chinese jujube (Fig. S2).

### Roles of the photoreceptors ZjPHYs and ZjCRY1 in Chinese jujube

Light is necessary for plant development, and plants deploy sensory photoreceptors such as phytochromes to capture light energy^20^. After capturing an incoming photon, activated phytochrome molecules must relay the information to nuclear genes that are poised to respond by directing appropriate adjustments in plant development^21^. Red/far-red/blue light sensing in higher plants is mediated by a diverse but structurally conserved group of soluble photoreceptors. In Arabidopsis, the phytochrome family consists of five members designated phyA to phyE^22^. In the present study, three phytochrome genes, *ZjPHYA*, *ZjPHYB*, and *ZjPHYC*, were identified in the jujube genome, and the expression of *ZjPHYA*, which mediates far-red-light stimulation, was higher during the vegetative growth stage than during the reproductive stage but gradually decreased during reproductive growth (Figs. 5 and 6a). *ZjPHYA* and *ZjPHYC* were also responsive to long- and short-day conditions (Fig. 6b). *ZjCRY1*, which encodes a blue light receptor, was also highly expressed in the juvenile period and in the early stage under protected cultivation (Figs. 5 and 6a).

### Roles of ZjPIF4 and ZjFT in Chinese jujube

PHYTOCHROME INTERACTING FACTOR 4 (PIF4), a core component of high-temperature signaling^18,19,23–25^, was identified as a molecular link connecting high-temperature signaling and stomatal development and revealed a direct mechanism by which the production of a specific cell lineage can be controlled by a broadly applicable environmental signaling factor^23^. The expression patterns of *ZjPIF4* were investigated under different conditions in an open field and under protected cultivation. In the open field, the expression of *ZjPIF4* increased at the later stage of flower development, and the temperature also increased during this period (in May). Under protected cultivation, *ZjPIF4* was highly expressed in early stages, in which the temperature ranged from 20 to 23 °C. Thus, the results under different conditions were consistent with each other, suggesting that *ZjPIF4* is responsive to increasing temperature. Previous studies have shown that high temperature induces the expression of *PIF4* at both the transcriptional and posttranscriptional levels^18,24,25^, and many PIF family transcription factors are well-established repressors of light signaling^19,26–28^ and interact with light-activated phytochromes^23,29–31^. In jujube, *ZjPIF4* interacted with the photoperiod-related protein ZjCO5 (Fig. 7b), which is consistent with the results of previous studies.

An unregulated photoperiod induces the expression of FLOWERING LOCUS T (FT) in the phloem companion cells (PCC) of leaves. The FT protein then acts as a florigen that is translocated to the shoot apical meristem (SAM)^32–36^. Based on the jujube genome annotation, there is only one FT gene in this species. ZjFT shared high identity with homologous genes in Rosacea species, e.g., 96.84% identity with MdFT (*Malus × domestica*, ACV92037.1), 93.68% identity with PbFT (Pyrus × bretschneideri, NP_001289254.1) and 91.95% identity with PpFT (*Prunus persica*, XP_007206002.1). Conserved domain analysis showed that ZjFT contains a phosphatidyl ethanolamine-binding protein (PEBP) domain, which is the conserved domain of FT. Taken together, all of these results showed that ZjFT is a homologous gene of FT.

As a key transcription factor in the bHLH family, PIF4 directly binds to the promoter region of FT and may also regulate FT at the protein level in Arabidopsis^37^. In the present study, ZjPIF4 was proven to interact with ZjFT at the protein level, which is consistent with the results of a previous study. Moreover, in Arabidopsis, PIF4 also interacts with DELLA proteins to integrate photoperiod–GA–circadian clock signals^38,39^. Thus, PIF4 likely has multiple functions at the transcriptional and translational levels, and its homologous genes in jujube and other fruit tree species are worthy of further research.

As shown in Figs. 4 and 5, the expression patterns of ZjPIF4 and ZjFT in the field and under protected cultivation were not different. In actuality, the growth period of the samples collected in protected cultivation (Fig. 5) occurred earlier than did that in the field (Figs. 1 and 4), and the later growth stages under protected cultivation were occurring just when the initial stages were occurring in the field. Thus, the expression patterns of ZjPIF4 and ZjFT at later stages in protected cultivation should be consistent with those at initial stages in the field, which was confirmed in this study.

*ZjFT* was highly expressed in bearing shoots before flowering in jujube (Fig. 5), and the expression pattern was similar to that of Arabidopsis^3^; thus this gene likely plays an important role in the transformation from vegetative growth to reproductive growth in Chinese jujube. However, the expression of *ZjFT* and *ZjPIF4* was higher on short days than on long days, and the jujube seedlings showed relatively early flowering under long-day conditions (Fig. 6b). Indeed, some related studies in Pharbitis^40^ and *Vitis vinifera* L.^41^ also showed similar results. In Pharbitis, the expression of *PnFT* increased when the night was longer than 11 h^40^. In *Vitis vinifera* L., shading treatment was beneficial for enhancing the expression of *VvPIF4*^41^. Thus, the expression of *ZjFT* and *ZjPIF4* in jujube might also be induced by night treatment under unique conditions or at specific developmental stages, and the detailed regulatory mechanism needs to be further elucidated.

## Material and methods

### Plant materials

Ten-year-old jujube (*Ziziphus jujuba* Mill. “Dongzao”) trees were grown at the experimental station of Hebei Agricultural University (HAU), Baoding, China. Roots, stems, leaves, flowers and young fruits were collected from three trees at 9:00 a.m. to 10:00 a.m. Bearing shoots from 0.5 mm to 20 mm were collected during flower bud differentiation, flower buds were harvested at the flower development stage, and flowers were collected while they were blooming.

Mother-bearing shoots and their phloem were harvested from ten-year-old Dongzao jujube trees under protected cultivation. The jujube trees were cultivated in a greenhouse, in which the indoor temperature was controlled at approximately 7–10 °C during the dormant period (approximately 20 days) and then was increased by 2–3 °C every week until the jujube trees sprouted. The temperature was maintained at 25–27 °C during the growth, flowering and fruiting periods, and the humidity remained at 60%–70%.

Jujube seedlings were sown in a laboratory with constant temperature and humidity, which were maintained at 23 °C and 70%, respectively. The plants were adapted to a 12 h light/12 h dark photoperiod for two weeks before treatment, with lights-on defined as zeitgeber time (ZT0) and lights-off defined as ZT12; ZT2 represents 2 h after the start of the light period. The seedlings were treated with long-day (14 h/10 h, 16 h/8 h, 18 h/6 h) and short-day (10 h/14 h, 8 h/16 h, 6 h/18 h) photoperiod treatments. A 12 h/12 h photoperiod treatment was used as the control. At 60 days after treatment, 15 seedlings were sampled in each treatment, and three biological replicates (5 seedlings mixed in one replicate) were collected at ZT2. Their flowering process was observed, and samples were stored at −80 °C for RNA extraction and expression analysis.

### Observation of the flowering process

When the perennial jujube trees began to sprout, we used Vernier calipers to measure dynamically and collect samples from the mother-bearing shoots at 9:00 a.m. (from April 5 to 18, 2019). The bearing shoots were subsequently cut into 0.5 mm, 1.0 mm, and 1.2 mm to 20 mm pieces (increments of 0.2 mm units) and then fixed with FAA solution. We then used paraffin to make permanent slices of the shoots^42^. After sealing solidly, the permanent air-dried slices were placed under a Zeiss light microscope to observe the morphological changes during flower bud development and were imaged by a ZEN universal imaging system.

To observe the process of flower development, we collected flower buds from the bud emergence stage to the blooming stage (from May 17 to June 22, 2019). According to the characteristics of flower development, we collected samples from 9:00 a.m to 10:00 a.m. The flower buds ranged from 500 to 2500 μm in size. We recorded the time and observed the morphological changes associated with flower opening under an XTJ-4400 stereomicroscope. CAM-MA imaging software was used to image the samples.

### Jujube seedlings used for phase transition

The tested materials were sown at the experimental station of HAU in 2018. Seedlings of JMS2×Xing16 offspring were used; there was a total of 68 plants under conventional management. Plant height and width were measured by a ruler, the base diameter and the initial diameter were measured by a vernier caliper, and the first flowering node and position were recorded. Bearing shoots were collected from jujube seedlings at 9:00 a.m. (on August 2018).

### Identification of flowering pathway genes

Based on the Arabidopsis flower network, the NCBI (http://www.ncbi.nlm.nih.gov/) and PFAM databases (http://pfam.sanger.ac.uk) were first used to identify the genes of various flowering pathways in the jujube genome. Homologous alignment was performed using the BLAST tool of NCBI to eliminate genes with inaccurate annotations. Moreover, a model (HMMER 3.0) was used to further search for all the flowering genes in the protein library^43^. The online tool ProtParam (http://web.expasy.org/protparam/) was used to predict the molecular weight (MW) and isoelectric point (pI) of each protein.

### Chromosomal location and gene duplication

Each identified gene was mapped to pseudochromosomes according to its coordinates on the jujube genome. Tandem duplications were investigated according to previous methods^44^. A circle graph was ultimately generated by TBtools (http://cj-chen.github.io/tbtools/).

### RNA isolation

According to the manufacturer’s instructions, total RNA was isolated from 200 mg of various tissues of jujube using an RNA Prep Pure Plant Kit (Tiangen, China). Using a TIAN Script First Strand cDNA Synthesis Kit (Tiangen, China), first-strand cDNA was synthesized from 2 μg of total RNA. The cDNA obtained was then diluted and stored at −20 °C for subsequent expression analyses.

### Gene expression analyses

Primers for semiquantitative RT-PCR and qRT-PCR were designed using Primer Premier 5.0, and expression patterns were assayed by semiquantitative RT-PCR. PCR was performed using the following program: initial denaturation at 95 °C for 3 min; 30 cycles at 95 °C for 15 s, 55 °C for 15 s, and 72 °C for 30 s; and a final extension cycle at 72 °C for 10 min^6^. The primers used in this study are listed in Table S5.

The expression of flowering-related genes at different developmental periods was examined using qRT-PCR (Bio-Rad iQ5). According to the instructions (Trans Start Top Green qPCR SuperMix), all reactions were performed in a 20 μL system consisting of 1 μL of cDNA, 10 μL of SYBR Green mix and each primer at 400 nM. The procedures were as follows: 94 °C for 30 s followed by 40 cycles of 94 °C for 5 s, 55 °C for 15 s and 72 °C for 15 s. All experiments were performed for three biological replicates, and each replicate was measured in triplicate. The specificity of the amplicon for each primer pair was verified by using melting curve analysis. *ZjACT* was used as a reference gene, and the relative expression levels of the tested genes were calculated by using the 2^−ΔΔCt^ method^6,45,46^.

### Prediction of protein–protein interactions

The interactions among photoperiod-related proteins in jujube were predicted by STRING (https://string-db.org/cgi/input.pl). Their orthologs in Arabidopsis were used as references.

### Yeast two-hybrid (Y2H) assays

Tested proteins were fused to the Gal4 DNA-binding domain (BD), and the screening proteins were fused to the Gal4 activation domain (AD). The AD-fused proteins and BD-fused proteins were amplified using the primers listed in Table S6 and then cloned into the pGADT7 and pGBKT7 vectors, respectively. The vectors were subsequently cotransformed into yeast strain AH109. *NdeI-SmaI* was used to digest the tested protein, and *EcoRI* was used to digest the candidate screening protein. The freshly transformed yeast colonies were resuspended in 10 μL of sterile deionized water, and 0.5 μL aliquots were spotted onto four selective media: synthetically defined (SD) media lacking leucine and tryptophan (−LW); media lacking leucine, tryptophan and histidine (−LWH); −LWH media supplemented with 7 mM 3-amino-1,2,4-triazole (3-AT, Sigma Aldrich) (−LWH+3AT); and media lacking leucine, tryptophan, adenine, and histidine (−LWAH). The growth conditions of the above yeast colonies were monitored after 3 days of incubation at 28 °C^47^.

## Supplementary information

Supplementary information

Supplementary information2

Supplementary information3

Supplementary information4

Supplementary information5

Supplementary information6

Supplementary information7

Supplementary information8
