# Supplementary material for: The regulatory pathways of distinct flowering characteristics in Chinese jujube
Source: Hortic Res. 2020 Aug 1;7:123. doi: 10.1038/s41438-020-00344-7 (PMC7395098; doi:10.1038/s41438-020-00344-7)
Supplement: Supplementary file 1 — Supplementary information [file 41438_2020_344_MOESM1_ESM.docx]

| **Stage** | **Length of fruiting branch(mm)** | **Differentiation characteristics** | **Differentiation time(h)** |
| --- | --- | --- | --- |
| Undifferentiated period | 0-2 | Flower buds appear in primitive bodies with a rounded growth point | 48 (Since the start of the germination) |
| Initial stage | 2-4 | The top of the growing point is flattened to form a flower primordium | 2-8 |
| Sepal differentiation | 4-8.6 | The top of the growth point is flat, and the sides are convex to form the sepal primordia | 36-48 |
| Petal differentiation | 8.6-9 | A protuberance is formed at the base of both sepals, and the petal primordia differentiates into petals | 144-168 |
| Stamen differentiation | 9-10 | Stamens protruding from both sides of petals | 38-48 |
| Carpel differentiation | 10 | The center of the flower bud primordium is slightly concave, and then the ovary and pistil are formed | 10-13 |

**Table S1 Corresponding relationship between length of jujube bearing shoots and flower bud differentiation**
