# Supplementary material for: The regulatory pathways of distinct flowering characteristics in Chinese jujube
Source: Hortic Res. 2020 Aug 1;7:123. doi: 10.1038/s41438-020-00344-7 (PMC7395098; doi:10.1038/s41438-020-00344-7)
Supplement: Supplementary file 2 — Supplementary information2 [file 41438_2020_344_MOESM2_ESM.doc]

**Table S2 Comparative analysis of homologous genes between jujube and other species**

| **Gene name** | ***Arabidopsis thaliana*** | ***Malus domestica*** | **Pyrus × bretschneideri** | ***Prunus persica*** | **Vitis vinifera** | ***Prunus mume*** |
| --- | --- | --- | --- | --- | --- | --- |
| *ZjCO1* | 61.22% | 73.93% | 77.86% | 78.73% | 76.12% | 77.66% |
| *ZjCO2* | 64.58% | 72.43% | 72.73% | 74.71% | 65.46% | 74.71% |
| *ZjCO3* | 53.99% | 43.56% | 40.31% | 68.21% | 64.23% | 67.18% |
| *ZjCO4* | 14.75% | 10.86% | 9.84% | 14.85% | 9.84% | 14.85% |
| *ZjCO5* | 58.12% | 73.03% | 72.79% | 78.52% | 11.89% | 78.52% |
| *ZjCO6* | 43.12% | 58.62% | 58.44% | 62.58% | 59.42% | 62.80% |
| *ZjCO7* | 40.80% | 55.42% | 53.86% | 60.99% | 53.33% | 60.99% |
| *ZjCO8* | 41.70% | 54.26% | 55.09% | 54.36% | 53.36% | 53.39% |
| *ZjCOP1* | 70.32% | 62.37% | 78.20% | 80.85% | 76.92% | 56.15% |
| *ZjGI* | 76.45% | 84.52% | 85.03% | 85.96% | 83.85% | 85.28% |
| *ZjPHYA* | 81.15% | 83.17% | 82.38% | 79.98% | 84.57% | 84.57% |
| *ZjPHYB* | 72.98% | 86.83% | 86.21% | 87.80% | 88.68% | 88.24% |
| *ZjPHYC* | 65.45% | 80.05% | 74.31% | 82.12% | 84.67% | 82.09% |
| *ZjCRY1* | 77.65% | 50.88% | 51.54% | 87.35% | 51.46% | 87.35% |
| *ZjAS1* | 69.52% | 83.43% | 85.11% | 87.15% | 86.87% | 85.91% |
| *ZjICE1* | 48.75% | 70.02% | 69.66% | 70.40% | 52.62% | 71.48% |
| *ZjNFYA1* | 41.14% | 68.14% | 68.89% | 72.65% | 44.73% | 72.93% |
| *ZjNFYB3* | 50.29% | 37.61% | 42.31% | 41.23% | 38.74% | 55.94% |
| *ZjNFYB5* | 49.40% | 49.47% | 61.78% | 51.69% | 69.01% | 56.89% |
| *ZjNFYC1* | 77.87% | 79.25% | 78.01% | 80.42% | 77.08% | 80.42% |
| *ZjNFYC2* | 57.45% | 82.67% | 82.44% | 20.70% | 81.88% | 86.43% |
| *ZjNFYC3* | 60.15% | 70.83% | 86.52% | 75.56% | 76.43% | 75.19% |
| *ZjNFYC9* | 57.47% | 78.63% | 77.86% | 84.23% | 80.92% | 84.23% |
| *ZjATX1* | 83.12% | 52.21% | 27.27% | 26.51% | 33.72% | 72.50% |
| *ZjATX2* | 59.53% | 72.23% | 72.50% | 73.99% | 66.31% | 73.81% |
| *ZjATXR7* | 34.26% | 47.71% | 47.70% | 49.55% | 49.09% | 34.12% |
| *ZjCLF* | 63.66% | 79.59% | 79.38% | 80.45% | 75.95% | 80.95% |
| *ZjEMF2* | 26.85% | 38.14% | 39.31% | 39.22% | 26.48% | 39.22% |
| *ZjFLC* | 40.00% | 47.37% | 49.51% | 50.00% | 12.23% | 49.35% |
| *ZjMSI1* | 90.09% | 93.85% | 94.80% | 95.27% | 95.99% | 95.27% |
| *ZjPHP* | 60.67% | 72.54% | 72.10% | 76.15% | 71.25% | 76.81% |
| *ZjVIP2* | 30.08% | 4.59% | 34.23% | 37.60% | 35.44% | 36.30% |
| *ZjAPRR5* | 37.41% | 58.32% | 60.31% | 60.88% | 26.08% | 57.81% |
| *ZjAPRR7* | 24.44% | 66.46% | 66.08% | 67.13% | 15.95% | 68.02% |
| *ZjELF3* | 25.25% | 31.05% | 33.84% | 36.67% | 32.12% | 44.25% |
| *ZjELF4* | 38.62% | 24.52% | 25.32% | 25.97% | 24.03% | 24.03% |
| *ZjLHY* | 36.68% | 52.50% | 63.20% | 60.41% | 58.93% | 68.97% |
| *ZjPCL1* | 50.88% | 68.00% | 69.54% | 72.89% | 31.61% | 31.15% |
| *ZjSLY1* | 75.96% | 88.28% | 88.59% | 88.91% | 88.91% | 88.59% |
| *ZjPIF4* | 34.77% | 58.30% | 57.49% | 57.69% | 57.38% | 54.71% |
| *ZjFPA* | 44.79% | 61.45% | 23.61% | 23.66% | 22.27% | 64.99% |
| *ZjFY* | 66.03% | 80.68% | 80.32% | 84.23% | 74.81% | 12.76% |
| *ZjFT* | 60.73% | 96.84% | 93.68% | 91.95% | 89.66% | 92.53% |
| *ZjSOC1* | 28.37% | 30.69% | 30.34% | 27.24% | 29.66% | 25.86% |
