# Supplementary material for: The regulatory pathways of distinct flowering characteristics in Chinese jujube
Source: Hortic Res. 2020 Aug 1;7:123. doi: 10.1038/s41438-020-00344-7 (PMC7395098; doi:10.1038/s41438-020-00344-7)
Supplement: Supplementary file 3 — Supplementary information3 [file 41438_2020_344_MOESM3_ESM.doc]

**Table S3 Index description of the end of juvenile of jujube seedlings in 2018 and 2019**

| **Cultivar** | **Blooming site** | **Average plant height(cm)** | **Average primordial node** | **Average physiological juvenile span(cm)** | **Average base diameter**  **(mm)** | **Average flowering diameter**  **(mm)** | **Flowering rate(%)** |
| --- | --- | --- | --- | --- | --- | --- | --- |
| JMS2×Xing16 | transitional secondary branch | 70.27±15.17 | 22±2.99 | 30.68±6.30 | 7.21±1.38 | 4.37±0.79 | 22% |

**2018**

2019

| **Cultivar** | **Blooming site** | **Average plant height (cm)** | **Average primordial node** | **Average physiological juvenile span (cm)** | **Average base diameter (mm)** | **Average flowering diameter (mm)** | **Flowering rate (%)** |
| --- | --- | --- | --- | --- | --- | --- | --- |
| JMS2×Xing16 | permanent secondary shoot | 89.08±18.66 | 29±3.89 | 39.69±12.84 | 12.35±2.39 | 7.04±1.91 | 100% |
