# Supplementary material for: The regulatory pathways of distinct flowering characteristics in Chinese jujube
Source: Hortic Res. 2020 Aug 1;7:123. doi: 10.1038/s41438-020-00344-7 (PMC7395098; doi:10.1038/s41438-020-00344-7)
Supplement: Supplementary file 4 — Supplementary information4 [file 41438_2020_344_MOESM4_ESM.doc]

**Table S4 Flowering statistics of jujube seedlings after photoperiod regulation treatments**

| **Treatment**  **(light/dark)** | **The number of flowering plants / Flowering rate** | | | |
| --- | --- | --- | --- | --- |
|  | **The 183^th^ day** | **The 191^th^ day** | **The 204^th^ day** | **The 216^th^ day** |
| CK(12h/12h) | 0 | 0 | 1/6.67% | 4/26.67% |
| LD(14h/10h) | 1/6.67% | 2/13.33% | 2/13.33% | 3/20% |
| LD(16h/08h) | 1/6.67% | 2/13.33% | 6/40% | 6/40% |
| LD(18h/06h) | 0 | 1/6.67% | 6/40% | 6/40% |
| SD(10h/14h) | 0 | 0 | 0 | 0 |
| SD(08h/16h) | 0 | 0 | 2/13.33% | 4/26.67% |
| SD(06h/18h) | 0 | 0 | 1/6.67% | 3/20% |

**Flowering rate indicates the percentage of the number of flowering plants in the total number of plants.**

**15 jujube seedlings are used as each treatment.**
