# Supplementary material for: The regulatory pathways of distinct flowering characteristics in Chinese jujube
Source: Hortic Res. 2020 Aug 1;7:123. doi: 10.1038/s41438-020-00344-7 (PMC7395098; doi:10.1038/s41438-020-00344-7)
Supplement: Supplementary file 5 — Supplementary information5 [file 41438_2020_344_MOESM5_ESM.doc]

| **Gene name**  **Table S5 Primer information for flowering-related genes in Chinese jujube** | **Pathway** | **Primers (5’-3’)** | **Product length(bp)** | **TM(℃)** |
| --- | --- | --- | --- | --- |
| ZjCO1 | Photoperiod | F:CGCGTTCCGATTCTACCCAT  R:ACCGGATTCAGCAACAACCA | 176 | 55.5 |
| ZjCO2 | Photoperiod | F:CACCGACGTGGATCCGTATT  R:TGTAGCCGTAGACGAAAGGC | 146 | 55 |
| ZjCO3 | Photoperiod | F:CTCCGCTAATCCTCTGGC  R:GGCTTCGGCTTCCTCAT | 163 | 56.2 |
| ZjCO4 | Photoperiod | F:AGAGGCAAGCGTGTTGAGAT  R:GAACCGGCCCTTCATACGAG | 168 | 55.5 |
| ZjCO5 | Photoperiod | F:TTCACGGCGCGAATTTTCTG  R:GCCTTTCTCGCACTTTCCAC | 161 | 56.7 |
| ZjCO6 | Photoperiod | F:CTGGAATGCTAATGGCTGTA  R:TAGGCAATGAACCAAGTGA | 167 | 56.7 |
| ZjCO7 | Photoperiod | F:CTCCCTCTGTGGTTAGTC  R:CCATCTTTGCCAGTTTT | 156 | 52 |
| ZjCO8 | Photoperiod | F:CGGCAAACAACCTATTC  R:CGATTCCATCAGACCC | 163 | 55.5 |
| ZjPHYA | Photoperiod | F:CCCAAGTATTTGCTATCC  R:ACCCAATGGTGCGTCT | 115 | 56.7 |
| ZjPHYB | Photoperiod | F:GCATTCCATTCCCACTACGC  R:TCGCACTTCACAAGGTCCAT | 205 | 60 |
| ZjPHYC | Photoperiod | F:TTTTGGCCCTCAGGAAAGCA  R:CCCCCTGAATGCGGGTAAAT | 155 | 54.1 |
| ZjNFYC1 | Photoperiod | F:CGAGCTCACCATCCGTTCTT  R:CAAACCGGCCTCATCCTTGA | 154 | 56.7 |
| ZjNFYC2 | Photoperiod | F:CGAGCTCACCATCCGTTCTT  R:CAAACCGGCCTCATCCTTGA | 122 | 56.2 |
| ZjNFYC3 | Photoperiod | F:GTGCCAAGGGAGGACTTGAA  R:CCATGTAGGGGTGAGACTGC | 199 | 57.7 |
| ZjNFYC9 | Photoperiod | F:TTCACGGCGCGAATTTTCTG  R:GCCTTTCTCGCACTTTCCAC | 181 | 60 |
| ZjNFYA1 | Photoperiod | F:TGCCACAAGCTAGAATGCCT  R:TTGCAAAGCGTCCTCCAGTA | 213 | 58.5 |
| ZjNFYB3 | Photoperiod | F:TGCTAAGGACGCCAAAGACA  R:CCTAGTGTTGCCATTGCCCA | 144 | 65 |
| ZjNFYB5 | Photoperiod | F:AGCTTCAAACTCCGAAGGGG  R:CACTTGACCGAGGCTTCACT | 183 | 55.5 |
| ZjCOP1 | Photoperiod | F:ATCAGCCATCCGCTTCATGT  R:AGGTGCGAACAGGCAGATTT | 145 | 56.2 |
| ZjAS1 | Photoperiod | F:GGGGTTTTGGGTTTTAGCCA  R:AGGTGCGAACAGGCAGATTT | 189 | 55 |
| ZjICE1 | Photoperiod | F:TGTCCCAGTTCATTGTCT  R:ATCATGGTGGAAAGCAG | 128 | 59.7 |
| ZjCRY1 | Photoperiod | F:GCTCCATGACCGGATACGAG  R:TCACGACCATCAGGGAGAGT | 168 | 55 |
| ZjGI | Photoperiod | F:CCCTCCAACATTCCTC  R:TACCCATTACACCACTACAC | 136 | 58.5 |
| ZjPHP | Vernalization | F:TACAGCACCTGTGGACCAAC  R:TGCCAGCAATCCTAGCAACA | 154 | 55.1 |
| ZjVIP2 | Vernalization | F:GGACCTCCTGGCATTGGATT  R:AGCTGACATTTGTTGCAGGC | 135 | 57.7 |
| ZjATX1 | Vernalization | F:GGGTTTGCACTGTGATGAATGT  R:TGTGCTTGCTGTCTTTCCAATC | 172 | 57.7 |
| ZjATXR7 | Vernalization | F:TATGAGGGTTTATCTACTGG  R:TGGCAAGGCACGAAAG | 191 | 53 |
| ZjATX2 | Vernalization | F:GCTGGTCTTTGCGTTGA  R:CCGAACATTGCCGAGT | 175 | 55.1 |
| ZjEMF2 | Vernalization | F:TAATTCGCAGGGTGTTGGCA  R:TGTTCATGGTGCAGGCATCTA | 165 | 55.7 |
| ZjCLF | Vernalization | F:GAAGCAGATAAGCGTGGAA  R:GCAACTCGCTTACTATTCCTTT | 101 | 57.7 |
| ZjMSI1 | Vernalization | F:TGGATGGTGCGTGTAGT  R:TTAGGTGTTGCGTTGATA | 148 | 51-55.7 |
| ZjFLC | Vernalization | F:AGGTCGCTCAAGATGGATGC  R:AGGTCATCTTCGATTGCCCG | 140 | 51 |
| ZjPIF4 | Ambient temperature | F:CTCTCAGAAAGGGAGAGATCG  R:TCATTGGTGCCATCCCACT | 173 | 55.5 |
| ZjLHY | Cirdian clock | F:CAACGAGAGCGATGGACAGA  R:TGTGGGAATAGCTGCACTGG | 276 | 60 |
| ZjPCL1 | Cirdian clock | F:CCGCAGCCTTATCACCG  R:GCGTTAGCCGACTCCAAG | 164 | 55 |
| ZjELF3 | Cirdian clock | F:GCCAGCCTTCAAACTA  R:CACAAACTGCTCCCATAA | 189 | 56.2 |
| ZjELF4 | Cirdian clock | F:CGAGCTCTGATCCAGCAAGT  R:ATCAACGCTGAGGAGGAGGA | 211 | 56.2 |
| ZjAPRR5 | Cirdian clock | F:AAATGGCCCCAAAGGTGACA  R:GAGCTCTGAGCATCACTCCC | 147 | 58.5 |
| ZjAPRR7 | Cirdian clock | F:ATTGGGTTCTGGCTCTCGAA  R:TGGTGGTTTAACTCCGGCAA | 169 | 57.1 |
| ZjFPA | Autonomous | F:AGAAGATGTCGGGTCGAGGA  R:ATGCGACGTTTTCGAGGTCT | 240 | 58.2 |
| ZjFY | Autonomous | F:TGGTGCAGAAATAGGCCAGG  R:AGCCAACCCAGGAGGAAATG | 162 | 60 |
| ZjSLY1 | Gibberellin | F:ATCCTCGTGCTCCTAA  R:GGCTGCTGCTGTTGTG | 244 | 59.7 |
| ZjFT | Flowering information integration factor | F:CTACAGGGGCAAGCTTTGGA  R:TGAAATTCTGACGCCACCCA | 141 | 62 |
| ZjSOC1 | Flowering information integration factor | F:TTTTGTGGTGGTCTTTC  R:CACTTGACGGCTTGTT | 234 | 55 |
