# Supplementary material for: The regulatory pathways of distinct flowering characteristics in Chinese jujube
Source: Hortic Res. 2020 Aug 1;7:123. doi: 10.1038/s41438-020-00344-7 (PMC7395098; doi:10.1038/s41438-020-00344-7)
Supplement: Supplementary file 6 — Supplementary information6 [file 41438_2020_344_MOESM6_ESM.doc]

**Table S6 Primer information for the target protein bait carrier**

| **Name** | **Primers (5'to3')** |
| --- | --- |
| ZjAS1-AD-F | ATGGAGGCCAGTGAAATGAAGGAGAGACAACGTTG |
| ZjAS1-AD-R | TTACCGGCCATTAGGCTCAGACCCGGGTGGAATTC |
| ZjAS1-BD-F | GGCCATGGAGGCCGAAATGAAGGAGAGACAACGTTG |
| ZjAS1-BD-R | TTACCGGCCATTAGGCTCAGGGATCCCCGGGAATTC |
| ZjFT-AD-F | GTACCAGATTACGCTCATATGATGCCTAGAGAAAGGGACCCTCTTGTTGTTGG |
| ZjFT-AD-R | CAGCTCGAGCTCGATGGATCCCTATCGTCTCCTTCCACCGGAGCCACTTTCCC |
| ZjFT-BD-F | TCAGAGGAGGACCTGCATATGATGCCTAGAGAAAGGGACCCTCTTGTTGTTGG |
| ZjFT-BD-R | CCGCTGCAGGTCGACGGATCCCTATCGTCTCCTTCCACCGGAGCCACTTTCCC |
| ZjCO5-AD-F | ATGGAGGCCAGTGAAATGATAAAGTGCGAGCTCTG |
| ZjCO5-AD-R | TCAATTGCTCTGATCTCTGCCACCCGGGTGGAATTC |
| ZjCO5-BD-F | GGCCATGGAGGCCGAAATGATAAAGTGCGAGCTCTG |
| ZjCO5-BD-R | TCAATTGCTCTGATCTCTGCCGGATCCCCGGGAATTC |
| ZjCO2-AD-F | ATGGAGGCCAGTGAAATGAAGTGTGAGCTCTGCGA |
| ZjCO2-AD-R | TCAACTTCTGCATTCCCTATCACCCGGGTGGAATTC |
| ZjCO2-BD-F | GGCCATGGAGGCCGAAATGAAGTGTGAGCTCTGCGA |
| ZjCO2-BD-R | TCAACTTCTGCATTCCCTATCGGATCCCCGGGAATTC |
| ZjPIF4-AD-F | GTACCAGATTACGCTCATATGGCCATGAATCACTGTATTCCTG |
| ZjPIF4-AD-R | CCGTATCGATGCCCACCCGGGGTTACAGTGAGGTATGAGCTCTTGC |
| ZjPIF4-BD-F | TCAGAGGAGGACCTGCATATGGCCATGAATCACTGTATTCCTG |
| ZjPIF4-BD-R | GCAGGTCGACGGATCCCCGGGGTTACAGTGAGGTATGAGCTCTTGC |
