# Supplementary figures and images for: The regulatory pathways of distinct flowering characteristics in Chinese jujube
Source: Hortic Res. 2020 Aug 1;7:123. doi: 10.1038/s41438-020-00344-7 (PMC7395098; doi:10.1038/s41438-020-00344-7)

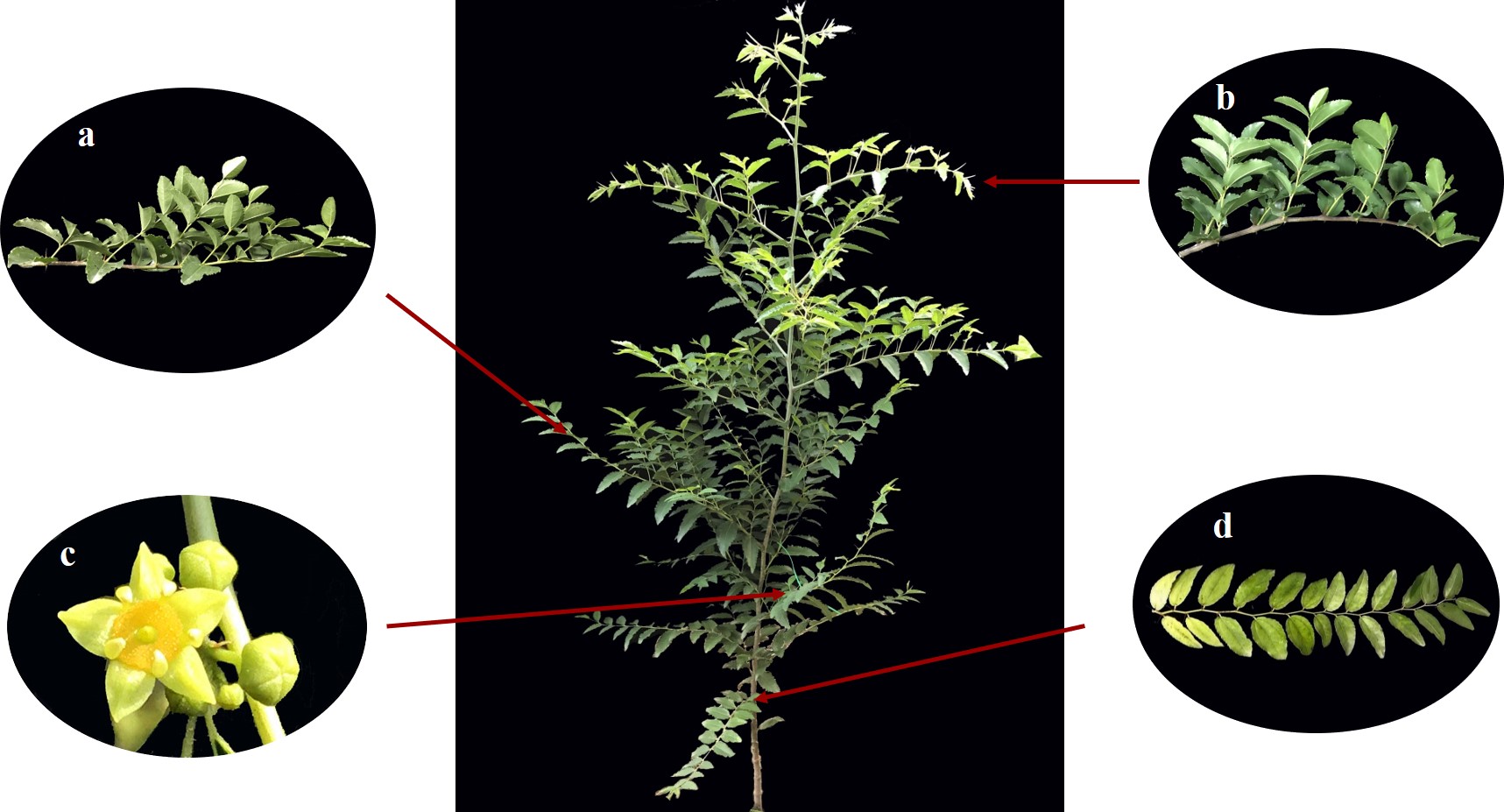

Supplement: Supplementary file 7 — Supplementary information7 [file 41438_2020_344_MOESM7_ESM.jpg]

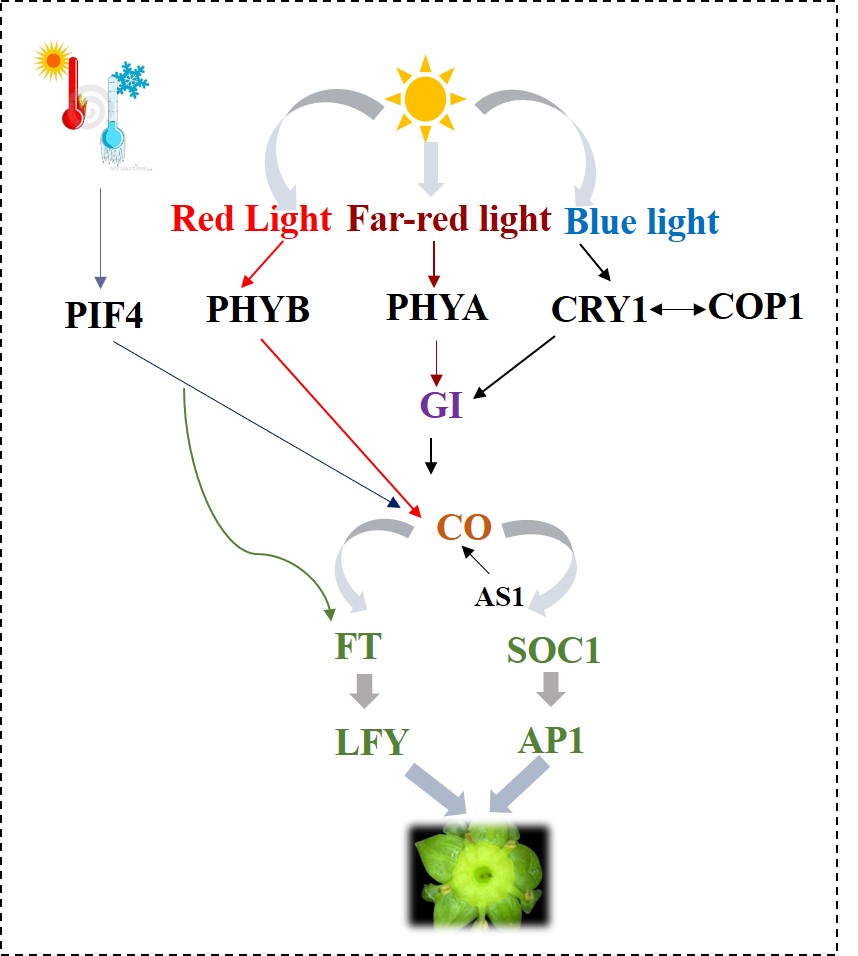

Supplement: Supplementary file 8 — Supplementary information8 [file 41438_2020_344_MOESM8_ESM.jpg]
